# Lower Density Selection Schemes via Small Universal Hitting Sets with Short Remaining Path Length

**DOI:** 10.1089/cmb.2020.0432

**Published:** 2021-04-20

**Authors:** Hongyu Zheng, Carl Kingsford, Guillaume Marçais

**Affiliations:** Computational Biology Department, Carnegie Mellon University, Pittsburgh Pennsylvania, USA.

**Keywords:** de Bruijn graph, depathing set, minimizers, sequence sketch, universal hitting set

## Abstract

Universal hitting sets (UHS) are sets of words that are unavoidable: every long enough sequence is hit by the set (i.e., it contains a word from the set). There is a tight relationship between UHS and minimizer schemes, where minimizer schemes with low density (i.e., efficient schemes) correspond to UHS of small size. Local schemes are a generalization of minimizer schemes that can be used as replacement for minimizer scheme with the possibility of being much more efficient. We establish the link between efficient local schemes and the minimum length of a string that must be hit by a UHS. We give bounds for the remaining path length of the Mykkeltveit UHS. In addition, we create a local scheme with the lowest known density that is only a log factor away from the theoretical lower bound.

## 1. Introduction

We study the problem of finding *Universal Hitting Sets* (UHS) (Orenstein et al., 2016). A UHS is a set of words, each of length *k*, such that every long enough string (say of length *L* or longer) contains as a substring element from the set. We call such a set a UHS for parameters *k* and *L*. They are sets of unavoidable words, that is, words that must be contained in any long strings, and we are interested in the relationship between the size of these sets and the length *L*.

More precisely, we say that a *k*-mer *a* (a string of length *k*) *hits* a string *S* if *a* appears as a substring of *S*. A set *A* of *k*-mers hits *S* if at least one *k*-mer of *A* hits *S*. A UHS for length *L* is a set of *k*-mers that hits every string of length *L*. Equivalently, the *remaining path length* of a universal set is the length of the longest string that is not hit by the set (L−1 here).

The study of UHS is motivated, in part, by the link between UHS and the common method of *minimizers* (Schleimer et al., 2003; Roberts et al., 2004a,b). The minimizer method is a way to sample a string for representative *k*-mers in a deterministic way by breaking a string into windows, each window containing *w k*-mers, and selecting in each window a particular *k*-mer (the “minimum *k*-mer,” as defined by a preset order on the *k*-mers). This method is used in many bioinformatic software programs (Ye et al., 2012; Grabowski and Raniszewski, 2013; Chikhi et al., 2015; Deorowicz et al., 2015; Jain et al., 2017) to reduce the amount of computation and improve run time (see Marçais et al., 2019 for usage examples). The minimizer method is a family of methods parameterized by the order on the *k*-mers used to find the minimum. The *density* is defined as the expected number of sampled *k*-mers per unit length of sequence. Depending on the order used, the density varies.

In general, a lower density (i.e., fewer sampled *k*-mers) leads to greater computational improvements and is therefore desirable. For example, a read aligner such as Minimap2 (Li and Birol, 2018) stores all the locations of minimizers in the reference sequence in a database. It then finds all the minimizers in a read and searches in the database for these minimizers. The locations of these minimizers are used as seeds for the alignment. Using a minimizer scheme with a reduced density leads to a smaller database and fewer locations to consider, hence an increased efficiency, while preserving the accuracy.

There is a two-way correspondence between minimizer methods and UHS: each minimizer method has a corresponding UHS, and a UHS defines a family of *compatible* minimizer methods (Marçais et al., 2017, 2018). This correspondence also links the remaining path length of a UHS and the window size of a compatible minimizer scheme: the remaining path length of the UHS is upper bounded by the number of bases in each window in the minimizer scheme (L≤w+k−1).

Moreover, the relative size of the UHS, defined as the size of UHS over the number of possible *k*-mers, provides an upper bound on the density of the corresponding minimizer methods: the density is no more than the relative size of the UHS. Precisely, $\frac{1}{w}\leq d\leq \frac{\left|U\right|}{\sigma^{k}}$, where *d* is the density, *U* is the UHS, *σ^k^* is the total number of *k*-mers on an alphabet of size *σ*, and *w* is the window length. In other words, the study of UHS with small size leads to the creation of minimizer methods with provably low density.

Local schemes (Mykkeltveit, 1972) and forward schemes are generalizations of minimizer schemes. These extensions are of interest because they can be used in place of minimizer schemes while sampling *k*-mers with lower density. In particular, minimizer schemes cannot have density close to the theoretical lower bound of 1∕w when *w* becomes large, while local and forward schemes do not suffer from this limitation (Marçais et al., 2018). Understanding how to design local and forward schemes with low density will allow us to further improve the computation efficiency of many bioinformatic algorithms.

The previously known link between minimizer schemes and UHS relied on the definition of an ordering between *k*-mers, and therefore is not valid for local and forward schemes that are not based on any ordering. Nevertheless, UHS play a central role in understanding the density of local and forward schemes.

Our first contribution is to describe the connection between UHS, local and forward schemes. More precisely, there are two connections: first, between the density of the schemes and the relative size of the UHS, and second, between the window size *w* of the scheme and the *remaining path length* of the UHS (i.e., the maximum length *L* of a string that does not contain a word from the UHS). This motivates our study of the relationship between the size of a UHS *U* and the remaining path length of *U*.

There is a rich literature on unavoidable word sets (Lothaire, 2002). The setting for UHS is slightly different for two reasons. First, we impose that all the words in the set *U* have the same length *k*, as a *k*-mer is a natural unit in bioinformatic applications. Second, the set *U* must hit any string of a given finite length *L*, rather than being unavoidable only by infinitely long strings.

Mykkeltveit (1972) answered the question of what is the size of a minimum unavoidable set with *k*-mers by giving an explicit construction for such a set. The *k*-mers in the Mykkeltveit set are guaranteed to be present in any infinitely long sequence, and the size of the Mykkeltveit set is minimum in the sense that for any set S with fewer *k*-mers, there is an infinitely long sequence that avoids S. On the contrary, the construction gives no indication on the remaining path length.

The DOCKS (Orenstein et al., 2016) and ReMuVal (DeBlasio et al., 2019) algorithms are heuristics to generate unavoidable sets for parameters *k* and *L*. Both of these algorithms use the Mykkeltveit set as a starting point. In many practical cases, the longest sequence that does not contain any *k*-mer from the Mykkeltveit set is much larger than the parameter *L* of interest (which for a compatible minimizer scheme corresponds to the window length). Therefore, the two heuristics extend the Mykkeltveit set to cover every *L*-long sequence. These greedy heuristics do not provide any guarantee on the size of the unavoidable set generated compared with the theoretical minimum size and are only computationally tractable for limited ranges of *k* and *L*.

Our second contribution is to give upper and lower bounds on the remaining path length of the Mykkeltveit sets. These are the first bounds on the remaining path length for minimum size sets of unavoidable *k*-mers.

Defining local or forward schemes with a density of O(1∕w) (i.e., within a constant factor of the theoretical lower bound) is not only of practical interest to improve the efficiency of existing algorithms, but it is also interesting for a historical reason. Both Roberts et al. (2004a) and Schleimer et al. (2003) used a probabilistic model to suggest that minimizer schemes have an expected density of 2∕w. Unfortunately, this simple probabilistic model does not correctly model the minimizer schemes outside of a small range of values for parameters *k* and *w*, and minimizers do not have an O(1∕w) density in general. Although the general question of whether a local scheme with O(1∕w) exists is still open, our third contribution is an almost-optimal forward scheme with density of O(ln(w)∕w) density. This is the lowest known density for a forward scheme, beating the previous best density of $O\left(\sqrt{w}∕w\right)$ (Marçais et al., 2018), and hinting that O(1∕w) might be achievable.

Understanding the properties of UHS and their many interactions with selection schemes (minimizer and forward and local schemes) is a crucial step toward designing schemes with lower density and improving the many algorithms using these schemes. In Section 2, we give an overview of the results, and in Section 3, we give detailed proofs. Further research directions are discussed in Section 4.

## 2. Results

### 2.1. Notation

#### 2.1.1. Universal hitting sets

Consider a finite alphabet Σ={0,…,σ−1} with σ≥2 elements. If a∈Σ, *a^k^* denotes the letter *a* repeated *k* times. We use $\Sigma^{k}$ to denote the set of strings of length *k* on alphabet Σ, and call them *k*-mers. If *S* is a string, S[n,l] denotes the substring starting at position *n* and of length *l*. For a *k*-mer $a\in \Sigma^{k}$ and an *l*-long string $S\in \Sigma^{l}$, we say “*a* hits *S*” if *a* appears as substring of *S* [a=S[i,k] for some *i*]. For a set of *k*-mers $A\subseteq \Sigma^{k}$ and $S\in \Sigma^{l}$, we say “*A* hits *S*” if there exists at least one *k*-mer in *A* that hits *S*. A set $A\subseteq \Sigma^{k}$ is a UHS for length *L* if *A* hits every string of length *L*.

#### 2.1.2. de Bruijn graphs

Many questions regarding strings have an equivalent formulation with graph terminology using *de Bruijn graphs*. The de Bruijn graph $B_{\Sigma ,k}$ on alphabet Σ and of order *k* has a node for every *k*-mer, and an edge (*u*, *v*) for every string of length k+1 with a prefix *u* and the suffix is *v*. There are *σ^k^* vertices and $\sigma^{k+1}$ edges in the de Bruijn graph of order *k*.

There is a one-to-one correspondence between strings and paths in $B_{\Sigma ,k}$: a path with *w* nodes corresponds to a string of L=w+k−1 characters. A UHS *A* corresponds to a *depathing set* of the de Bruijn graph: a UHS for *k* and *L* intersects with every path in the de Bruijn graph with w=L−k+1 vertices. We say “*A* is a (α,l)-UHS” if *A* is a set of *k*-mers that is a UHS, with relative size $\alpha =|A|∕\sigma^{k}$ and hits every walk of *l* vertices (and therefore every string of length L=l+k−1).

A *de Bruijn sequence* is a particular sequence of length $\sigma^{k}+k-1$ that contains every possible *k*-mer once and only once. Every de Bruijn graph is Hamiltonian and the sequence spelled out by a Hamiltonian tour is a de Bruijn sequence.

#### 2.1.3. Selection schemes

A *local scheme* (Schleimer et al., 2003) is a method to select positions in a string. A local scheme is parameterized by a *selection function f*. It works by looking at every *w*-mer of the input sequence *S*: S[0,w],S[1,w],…, and selecting in each window a position according to the selection function *f*. The selection function selects a position in a window of length *w*, that is, it is a function $f:\Sigma^{w}\to \left[0:w-1\right]$. The output of a forward scheme is a set of selected positions: {i+f(S[i,w])|0≤i<|S|−w}.

A *forward scheme* is a local scheme with a selection function such that the selected positions form a nondecreasing sequence. That is, if $\omega_{1}$ and $\omega_{2}$ are two consecutive windows in a sequence *S*, then $f\left(\omega_{2}\right)\geq f\left(\omega_{1}\right)-1$.

A *minimizer scheme* is a scheme where the selection function takes in the sequence of *w* consecutive *k*-mers and returns the “minimum” *k*-mer in the window (hence the name minimizers). The minimum is defined by a predefined order on the *k*-mers (e.g., lexicographic order) and the selection function is $f:\Sigma^{w+k-1}\to \left[0:w-1\right]$.

**FIG. 1.**
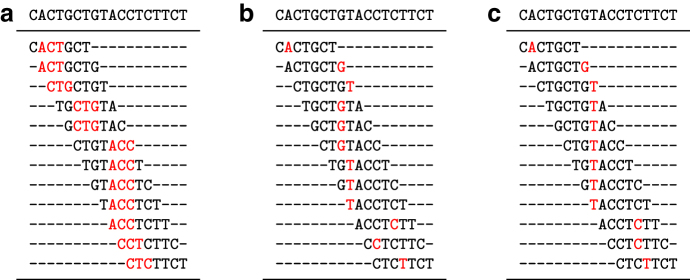
**(a)** Example of selecting minimizers with k=3, w=5, and the lexicographic order (i.e., AAA<AAC<AAG<…<TTT). The top line is the input sequence, each subsequent line is a 7-bases long window (the number of bases in a window is w+k−1=7) with the minimum 3-mer highlighted. The positions {1, 2, 5, 9, 10, 11} are selected for a density d=6∕(18−3+1)=0.375. **(b)** On the same sequence, an example of a selection scheme for w=7 (and k=1 because it is a selection scheme, hence the number of bases in a window is also *w*). The set of positions selected is {1, 6, 7, 8, 11, 13, 14}. This is not a forward scheme as the sequence of selected position is not decreasing. **(c)** A forward selection scheme for w=7 with selected positions {1, 7, 8, 12, 13}. Like the minimizer scheme, the sequence of selected positions is nondecreasing.

See Figure 1 for examples of all three schemes. The local scheme concept is the most general as it imposes no constraint on the selection function, while a forward scheme must select positions in a nondecreasing way. A minimizer scheme is the least general and also selects positions in a nondecreasing way.

Local and forward schemes were originally defined with a function defined on a window of *w k*-mers, $f:\Sigma^{w+k-1}\to \left[0:w-1\right]$, similarly to minimizers. Selection schemes are schemes with k=1, and have a single parameter *w* as the word length. While the notion of *k*-mer is central to the definition of the minimizer schemes, it has no particular meaning for a local or forward scheme: these schemes select positions within each window of a string *S*, and the sequence of the *k*-mers at these positions is no more relevant than a sequence elsewhere in the window to the selection function.

There are multiple reasons to consider selection schemes. First, they are slightly simpler as they have only one parameter, namely the window length *w*. Second, in our analysis, we consider the case where *w* is asymptotically large, therefore w≫k and the setting is similar to having k=1. Finally, this simplified problem still provides information about the general problem of local schemes. Suppose that *f* is the selection function of a selection scheme, for any k>1 we can define $g_{k}:\Sigma^{w+k-1}\to \left[0,w-1\right]$ as $g_{k}\left(\omega \right)=f\left(\omega \left[0,w\right]\right)$. That is, *g_k_* is defined from the function *f* by ignoring the last k−1 characters in a window. The functions *g_k_* define proper selection functions for local schemes with parameters *w* and *k*, and because exactly the same positions are selected, the density of *g_k_* is equal to the density of *f*. In the following sections, unless noted otherwise, we use forward and local schemes to denote forward and local selection schemes.

#### 2.1.4. Density

Because a local scheme on string *S* may pick the same location in two different windows, the number of selected positions is usually less than |S|−w+1. The *particular density* of a scheme is defined as the number of distinct selected positions divided by |S|−w+1 (Fig. 1). The *expected density*, or simply the *density*, of a scheme is the expected density on an infinitely long random sequence. Alternatively, the expected density is computed exactly by computing the particular density on any de Bruijn sequence of order ≥2w−1. In other words, a de Bruijn sequence of large enough order “looks like” a random infinite sequence with respect to a local scheme (see Marçais et al., 2017 and Section 3.1).

### 2.2. Main results

The density of a local scheme is in the range [1∕w,1], as 1∕w corresponds to selecting exactly one position per window, and 1 corresponds to selecting every position. Therefore, the density goes from a low value with a constant number of positions per window [density is O(1∕w), which goes to 0 when *w* gets large], to a high with constant value [density is Ω(1)] where the number of positions per window is proportional to *w*. When the minimizers and winnowing schemes were introduced, both articles used a simple probabilistic model to estimate the expected density to 2∕(w+1), or about 2 positions per window. Under this model, this estimate is within a constant factor of the optimal, O(1∕w).

Unfortunately, this simple model properly accounts for the minimizer behavior only when *k* and *w* are small. For large *k*—that is, k≫w—it is possible to create an almost-optimal minimizer scheme with a density ∼1∕w. More problematic, for large *w*—that is, w≫k—and for all minimizer schemes, the density becomes constant [Ω(1)] (Marçais et al., 2018). In other words, minimizer schemes cannot be optimal or within a constant factor of optimal for large *w*, and the estimate of 2∕(w+1) is very inaccurate in this regime.

This motivates the study of forward schemes and local schemes. It is known that there exist forward schemes with a density of $O\left(1∕\sqrt{w}\right)$ (Marçais et al., 2018). This density is not within a constant factor of the optimal density but at least shows that forward and local schemes do not have constant density such as minimizer schemes for large *w* and that they can have much lower density.

#### 2.2.1. Connection between UHS and selection schemes

In the study of selection schemes, as for minimizer schemes, UHS play a central role. We describe the link between selection schemes and UHS, and show that the existence of a selection scheme with low density implies the existence of a UHS with a small relative size.

**Theorem 1.** *Given a local scheme f on w-mers with density d_f_*, *we can construct a* $\left(d_{f},w\right)-UHS$ *on* (2w−1)*-mers*. *If f is a forward scheme, we can construct a* $\left(d_{f},w\right)-UHS$ *on* (w+1)*-mers*.

#### 2.2.2. Almost-optimal relative size UHS for linear path length

Conversely, because of their link to forward and local selection schemes, we are interested in UHS with remaining path length O(w). Necessarily a universal hitting hits any infinitely long sequences. On de Bruijn graphs, a set hitting every infinitely long sequence is a *decycling set*: a set that intersects with every cycle in the graph. In particular, a decycling set must contain an element in each of the cycles obtained by the rotation of the *w*-mers (e.g., cycle of the type 001→010→100→001). The number of these rotation cycles is known as the “necklace number” $N_{\sigma ,w}=\frac{1}{n}\sum_{d|w}\varphi \left(d\right)\sigma^{w∕d}=O\left(\sigma^{w}∕w\right)$ (Golomb, 2014), where φ(d) is the Euler's totient function.

Consequently, the relative size of a UHS, which contains at least one element from each of these cycles, is lower bounded by O(1∕w). The smallest previously known UHS with O(w) remaining path length has a relative size of $O\left(\sqrt{w}∕w\right)$ (Marçais et al., 2018). We construct a smaller UHS with relative size O(ln(w)∕w):

**Theorem 2.** *For every sufficiently large w, there is a forward scheme with density of* O(ln(w)∕w) *and a corresponding* (O(ln(w)∕w),w)*-UHS.*

#### 2.2.3. Remaining path length bounds for the Mykkeltveit sets

Mykkeltveit (1972) gave an explicit construction for a decycling set with exactly one element from each of the rotation cycles, and thereby proved a long-standing conjecture (Golomb, 2014) that the minimal size of decycling sets is equal to the necklace number. Under the UHS framework, it is natural to ask what the remaining path length for Mykkeltveit sets is. Given that the de Bruijn graph is Hamiltonian, there exist paths of length exponential in *w*: the Hamiltonian tours have *σ^w^* vertices. Nevertheless, we show that the remaining path length for Mykkeltveit sets is upper and lower bounded by polynomials of *w*:

**Theorem 3.** *For sufficiently large w, the Mykkeltveit set is a* $\left(N_{\sigma ,w}∕\sigma^{w},g\left(w\right)\right)$*-UHS, having the same size as minimal decycling sets, while* $c_{1}w^{2}\leq g\left(w\right)\leq c_{2}w^{3}$ *for some constants c*_1_ *and c*_2_.

## 3. METHODS AND PROOFS

For length reasons, several parts of the proof are found in the Supplementary Material.

### 3.1. UHS from selection schemes

#### 3.1.1. Contexts and densities of selection schemes

We derive another way of calculating densities of selection schemes based on the idea of *contexts*.

Recall a local scheme is defined as a function $f:\Sigma^{w}\to \left[0,w-1\right]$. For any sequence *S* and scheme *f*, the set of selected locations is {f(S[i,w])+i} and the density of *f* on the sequence is the number of selected locations divided by |S|−w+1. Counting the number of distinct selected locations is the same as counting the number of *w*-mers S[i,w] such that *f* picks a new location from all previous *w*-mers. *f* can pick identical locations on two *w*-mers only if they overlap, so intuitively, we only need to look back (w−1) windows to check if the position is already picked. Formally, *f* picks a new position in window S[i,w] if and only if f(S[i,w])+i≠f(S[i−d,w])+(i−d) for all 1≤d≤w−1.

For a location *i* in sequence *S*, the context at this location is defined as $c_{i}=S\left[i-w+1,2w-1\right]$, a (2w−1)-mer whose last *w*-mer starts at *i*. Whether *f* picks a new position in S[i,w] is entirely determined by its context, as the conditions only involve *w*-mers as far back as S[i−w+1,w], which are all included in the context. This means that instead of counting selected positions in *S*, we can count the contexts *c* satisfying f(c[w−1,w])+w−1≠f(c[j,w])+j for all 0≤j≤w−2, which are the contexts such that *f* on the last *w*-mer of *c* picks a new location. We denote by $S_{f}\subset \Sigma^{2w-1}$ the set of contexts that satisfy this condition.

**Definition 1.** For given w and local selection scheme $f:\Sigma^{w}\to \left[0,w-1\right]$, $S_{f}=\left\{c\in \Sigma^{2w-1}|\forall 0\leq i\leq w-2,f\left(c\left[w-1,w\right]\right)+\left(w-1\right)\neq f\left(c\left[i,w\right]\right)+i\right\}$ is a subset of $\Sigma^{2w-1}$.

The expected density of *f* is computed as the number of selected positions over the length of the sequence for a random sequence, as the sequence becomes infinitely long. For a sufficiently long random sequence (|S|≫w), the distribution of its contexts converges to a uniform random distribution over (2w−1)-mers. Because the distribution of these contexts is exactly equal to the uniform distribution on a circular de Bruijn *S* sequence of order at least 2w−1, we can calculate the expected density of *f* as the density of *f* on *S*, or as $|S_{f}|∕\sigma^{2w-1}$.

#### 3.1.2. UHS from selection schemes

The set $S_{f}$ over (2w−1)-mers is the UHS needed for Theorem 1. Intuitively, it is a UHS with remaining path length of at most w−1, because one location must be picked every *w* window, meaning there is a window that picked a new location. The context that is prefix of this window is in $S_{f}$ by definition.

**Lemma 1.** $S_{f}$ *is a UHS with remaining path length of at most* w−1.

*Proof.* By contradiction, assume there is a path of length *w* in the de Bruijn graph of order (2w−1), say $\left\{c_{0},c_{1},\cdots ,c_{w-1}\right\}$, that avoids S. We construct the sequence *S*′ corresponding to the path: $S\prime \in \Sigma^{3w-2}$ such that $S\prime \left[i,2w-1\right]=c_{i}$.

Since $c_{w-1}\notin S$ and S′ include $c_{w-1}$, it means *f* on the last *w*-mer of $c_{w-1}$ (which is S′[2w−2,w]) picks a location that has been picked before on S′. The coordinate *l* of this selection in S′ satisfies l≥2w−2. As 0≤f(x)≤w−1, the first *w*-mer S′[m,w] in S′ such that *f* picks S′[l] (i.e., m+f(S′[m,w])=l) satisfies m≥w−1. The context $c_{m-w+1}=S\prime \left[m-\left(w-1\right),2w-1\right]$ then satisfies that a new location *l* is picked when *f* is applied to its last *w*-mer, and by definition $c_{m-w+1}\in S$, contradiction.

This results is also a direct consequence of the definition of S. An alternative direct proof is available in Supplementary Section S1.

When *f* is a forward scheme, to determine if a new location is picked in a window, looking back one window is sufficient. This is because if we do not pick a new location, we have to pick the same location as in the last window. This means the context with two *w*-mers, or as a (*w* + 1)-mer, is sufficient, and our other arguments involving contexts still hold. Combining the pieces, we prove the following theorem:

**Theorem 4.** *Given a local scheme f on w-mers with density d_f_*, *we can construct a* (*d_f_*, *w*) − *UHS on* (2*w* − 1)*-mers. If f is a forward scheme, we can construct a* (*d_f_*, *w*) − *UHS on* (*w* + 1)*-mers*.

### 3.2. Forbidden word depathing set

#### 3.2.1. Construction and path length

In this section, we construct a set that is (O(ln(w)∕w),w)−UHS.

**Definition 2** (Forbidden Word UHS). Let $d=\left\lfloor \log_{\sigma }\left(w∕\ln\left(w\right)\right)\right\rfloor -1$. Define ${ℱ}_{\sigma ,w}$ as the set of *w*-mers that satisfies either of the following clauses: (1) 0^*d*^ is the prefix of *x* (2) 0^*d*^ is not a substring of *x*.

We assume that *w* is sufficiently large such that d≥1.

**Lemma 2.** *The longest remaining path in the de Bruijn graph of order w after removing* ${ℱ}_{\sigma ,w}$ *is* w−d.

*Proof.* Let $\left\{x_{0},x_{1},\cdots ,x_{w-d}\right\}$ be a path of length w−d+1 in the de Bruijn graph. If *x*_0_ does not have a substring equal to 0^*d*^, it is in ${ℱ}_{\sigma ,w}$. Otherwise, let *c* be the index such that $x_{0}\left[c,d\right]=0^{d}$. Since c≤w−d, $x_{c}\left[0,d\right]=0^{d}$ and *x_c_* is in ${ℱ}_{\sigma ,w}$.

On the contrary, let $S=1^{w-d}0^{d}1^{w-d-1}\in \Sigma^{2w-d-1}$ and $x_{i}=S\left[i,w\right]$ for 0≤i<w−d. None of $\left\{x_{i}\right\}$ is in ${ℱ}_{\sigma ,w}$, meaning there is a path of length w−d in the remaining graph.

The number of *w*-mer satisfying clause 1 is $\sigma^{w-d}=O\left(\ln\left(w\right)\sigma^{w}∕w\right)$. For the rest of this section, we focus on counting *w*-mers satisfying clause 2 in Definition 2, that is, the number of *w*-mers not containing 0^*d*^.

#### 3.2.2. Number of *w*-mers not containing 0^*d*^

We construct a finite state machine (FSM) that recognizes 0^*d*^ as follows. The FSM consists of d+1 states labeled “0” to “d,” where “0” is the initial state and “d” is the terminal state. The state “*i*” with 0≤i≤d−1 means that the last *i* characters were *0* and d−i more zeroes are needed to match 0^*d*^. The terminal state “d” means that we have seen a substring of *d* consecutive zeroes. If the machine is at nonterminal state “i” and receives the character 0, it moves to state “i+1,” otherwise it moves to state “0”; once the machine reaches state “d,” it remains in that state forever.

Now, assume we feed a random *w*-mer to the FSM. The probability that the machine does not reach state “d” for the input *w*-mer is the relative size of the set of *w*-mer satisfying clause 2. Denote $p_{k}\in {ℛ}^{d}$ such that $p_{k}\left(j\right)$ is the probability of feeding a random *k*-mer to the machine and ending up in state “j,” for 0≤j<d (note that the vector does not contain the probability for the terminal state “d”). The answer to our problem is then ${∥p_{w}∥}_{1}=\sum_{i=0}^{d-1}p_{w}\left(i\right)$, that is, the sum of the probabilities of ending at a nonterminal state.

Define μ=1∕σ. Given that a randomly chosen *w*-mer is fed into the FSM, that is, each base is chosen independently and uniformly from Σ, the probabilities of transition in the FSM are: “i” → “i + 1” with probability *μ*, “i” → “0” with probability 1−μ. The probability matrix to not recognize 0^*d*^ is a d×d matrix, as we discard the row and column associated with terminal state “d”:

$$A_{d}={\left[\begin{array}{ccccc} 1-\mu & 1-\mu & \ldots & 1-\mu & 1-\mu \\ \mu & 0 & \ldots & 0 & 0\\ 0 & \mu & \ldots & 0 & 0\\ \vdots & \vdots & \ddots & \vdots & \vdots \\ 0 & 0 & \ldots & \mu & 0 \end{array}\right]}_{d\times d}=\left[\begin{array}{cc} \left(1-\mu \right)1_{d-1}^{T} & 1-\mu \\ \mu I_{d-1} & 0_{d-1} \end{array}\right]$$

Starting with $p_{0}=\left(1,0,\ldots ,0\right)\in {ℛ}^{d}$ as initially no sequence has been parsed and the machine is at state “0” with probability 1, we can compute the probability vector *p_w_* as $p_{w}=A_{d}p_{w-1}=A_{d}^{w}p_{0}$.

#### 3.2.3. Bounding ${∥p_{w}∥}_{1}$

We start by deriving the characteristic polynomial $p_{A_{d}}\left(\lambda \right)$ of *A_d_* and its roots, which are the eigenvalues of *A_d_*:

**Lemma 3.**

$$p_{A_{d}}\left(\lambda \right)=\det\left(A_{d}-\lambda I\right)=\left\{\begin{array}{cc} {\left(-1\right)}^{d}\frac{\lambda^{d+1}-\lambda^{d}-\mu^{d+1}+\mu^{d}}{\lambda -\mu } & \lambda \neq \mu \\ {\left(-\mu \right)}^{d-1}\left(\left(1-\mu \right)d-\mu \right) & \lambda =\mu \end{array}\right.$$

*Proof.* The characteristic polynomial of *A_d_* satisfies the following recursive formula, obtained by expanding the determinant over the first column and using the linearity of the determinant: 
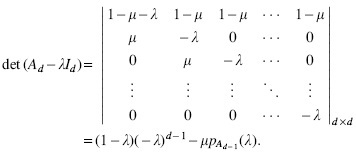

For d=1, we have $p_{A_{1}}\left(\lambda \right)=1-\mu -\lambda$. Assuming λ≠μ for now, we repeatedly expand the recursive formula to obtain a closed-form formula for $p_{A_{d}}\left(\lambda \right)$: 
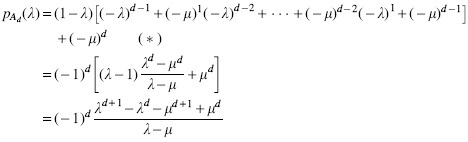
.

Now we fix *d* and focus on the polynomial $f_{d}\left(\lambda \right)=\lambda^{d+1}-\lambda^{d}-\mu^{d+1}+\mu^{d}$. Since this is a polynomial of degree d+1, it has d+1 roots and except for *μ*, which is a root of *f_d_* but not of $p_{A_{d}}$, *f_d_* and $p_{A_{d}}$ have the same roots.

**Lemma 4.** *For sufficiently large d,* $f_{d}\left(\lambda \right)$ *has a real root λ*_0_ *satisfying* $1-\mu^{d}<\lambda_{0}<1-\mu^{d+1}$.

*Proof.* We show *f_d_* has opposite signs on the lower and upper bound of this inequality for sufficiently large *d*. 
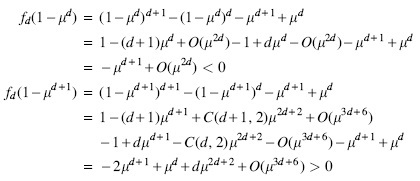

For the last line, if σ=2 the first two terms cancel out and $d\mu^{2d+2}$ becomes dominant and positive, otherwise $\mu^{d}=\sigma \mu^{d+1}>2\mu^{d+1}$. Since *f_d_* is polynomial, *f_d_* is continuous and thus has a root between $1-\mu^{d}$ and $1-\mu^{d+1}$.

**Lemma 5.** *Let* $s=\mu ∕\lambda_{0}$. $\nu_{0}=\left(1,s,s^{2},\cdots ,s^{d-1}\right)$ *is the right eigenvector of A_d_ corresponding to eigenvalue* $\lambda_{0}$, *and* ${∥\nu_{0}∥}_{1}<3$ *for sufficiently large d.*

*Proof.* For the first part, we need to verify $A_{d}\nu_{0}=\lambda_{0}\nu_{0}$. For indices 1≤i<d,(Adν0)_i_=μ(ν_0_)_i-1_=μs^i-1^=λ_0_s^i^=(λ_0_ν_0_)_i_. For the first element in the vector, we have: 
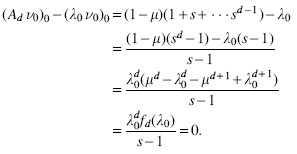

This verifies $A_{d}\nu_{0}=\lambda_{0}\nu_{0}$. For the second part, note that for sufficiently large *d* we have $\lambda_{0}>1-\mu^{d}>0.9$ and since μ≤0.5, we have $s=\mu ∕\lambda_{0}<2∕3$. Every element of $\nu_{0}$ is positive, so ${∥\nu_{0}∥}_{1}=\sum_{i=0}^{d-1}s^{i}<\sum_{i=0}^{\infty }s^{i}=1∕\left(1-s\right)<3$.

**Lemma 6.** ${∥p_{w}∥}_{1}={∥A_{d}^{w}p_{0}∥}_{1}=O\left(1∕w\right)$.

*Proof.* Let $\eta_{0}=\nu_{0}-p_{0}=\left(0,s,s^{2},\cdots ,s^{d-1}\right)$, where $s=\mu ∕\lambda_{0}$ from last lemma. Because $\lambda_{0}>0$, the elements of $\eta_{0}$ and *A_d_* are all non-negative, then the elements of $A_{d}^{w}\eta_{0}$ and $\lambda_{0}\eta_{0}$ are also non-negative. Now, recall that $d=\left\lfloor \log_{\sigma }\left(w∕\ln\left(w\right)\right)\right\rfloor -1$, which implies that $\mu^{d+1}\geq \ln\left(w\right)∕w$. 
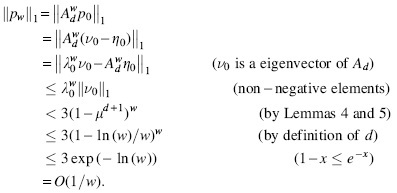

This lemma implies that the relative size for the set ${ℱ}_{\sigma ,w}$ is dominated by the *w*-mers satisfying clause 1 of Definition 2 and ${ℱ}_{\sigma ,w}$ is of relative size O(ln(w)∕w). This completes the proof that ${ℱ}_{\sigma ,w}$ is (O(ln(w)∕w),w)−UHS.

### 3.3. Construction of the Mykkeltveit sets

In this section, we construct the Mykkeltveit set ${ℳ}_{\sigma ,w}$ and prove some important properties of the set. We start with the definition of the Mykkeltveit embedding of the de Bruijn graph.

**Definition 3** (Modified Mykkeltveit Embedding). For a *w*-mer *x*, its embedding in the complex plane is defined as $P\left(x\right)=\sum_{i=0}^{w-1}x_{i}r_{w}^{i+1}$, where *r_w_* is a *w*th root of unity, $r_{w}=e^{2\pi i∕w}$.

Intuitively, the position of a *w*-mer *x* is defined as the following center of mass. The *w* roots of unity form a circle in the complex plane, and a weight equal to the value of the base *x_i_* is set at the root $r_{w}^{i+1}$. The position of *x* is the center of mass of these *w* points and associated weights. Originally, Mykkeltveit defined the embedding with weight $r_{w}^{i}$ (Mykkeltveit, 1972). This extra factor of *r_w_* in our modified embedding rotates the coordinate and is instrumental in the proof.

Define the *successor function* $S_{a}\left(x\right)=x_{1}x_{2}\cdots x_{w-1}a$, where a∈Σ. The successor function gives all the neighbors of *x* in the de Bruijn graph. A *pure rotation* of *x* is the particular neighbor $R\left(x\right)=S_{x_{0}}\left(x\right)$, that is, the sequence of R(x) is a left rotation of *x*.

We focus on a particular kind of cycle in the de Bruijn graph. A *pure cycle* in the de Bruijn graph, also known as *conjugacy class*, is the sequence of *w*-mers obtained by repeated rotation: $\left(x,R\left(x\right),R^{2}\left(x\right),\ldots \right)$. Each pure cycle consists of *w* distinct *w*-mer s, unless $x_{0}x_{1}\cdots x_{w-1}$ is periodic, and in this case, the size of the cycle is equal to its shortest period.

The embeddings from pure rotations satisfy a curious property:

**Lemma 7** (Rotations and Embeddings). P(R(x)) *on the complex plane is* P(x) *rotated clockwise around origin by* 2π∕w. $P\left(S_{a}\left(x\right)\right)$ *is* P(R(x)) *shifted by* $\delta =a-x_{0}$ *on the real part, with the imaginary part unchanged.*

*Proof.* By Definition 3 and the definition of successor function $S_{a}\left(x\right)$: 
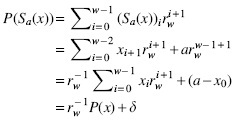

Note that for pure rotations δ=0, and $r_{w}^{-1}P\left(x\right)$ is exactly P(x) rotated clockwise by 2π∕w.

**FIG. 2.**
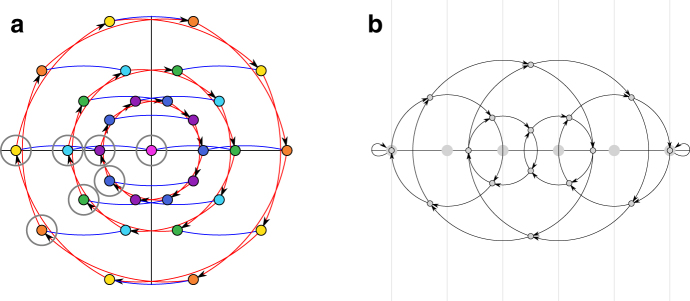
**(a)** Mykkeltveit embedding of the de Bruijn graph of order 5 on the binary alphabet. The nodes of a conjugacy class have the same color and form a circle (there is more than one class per circle). The pure rotations are represented by the red edges. A nonpure rotation $S_{a}\left(x\right)$ is a red edge followed by a horizontal shift (blue edge). The set of nodes circled in gray is the Mykkeltveit set. **(b)** Weight-in embedding of the same graph. Multiple *w*-mers map to the same position in this embedding and each circle represents a conjugacy class. The gray dots on the horizontal axis are the *w* centers of rotations and the vertical gray lines going through the centers separate the space in subregions of interest.

The range for δ is [−σ+1,σ−1]. In particular, δ can be negative. In a pure cycle, either all *w*-mers satisfy P(x)=0, or they lie equidistant on a circle centered at origin. Figure 2a shows the embeddings and pure cycles of 5-mers. It is known that we can partition the set of all *w*-mers into $N_{\sigma ,k}$ disjoint pure cycles. This means any decycling set that breaks every cycle of the de Bruijn graph will be at least this large. We now construct our proposed depathing set with this idea in mind.

**Definition 4** (Mykkeltveit set). We construct the Mykkeltveit set ${ℳ}_{\sigma ,w}$ as follows. Consider each conjugacy class, we will pick one *w*-mer from each of them by the following rule:

1. If every *w*-mer in the class embeds to the origin, pick an arbitrary one.
2. If there is one *w*-mer *x* in the class such that Re(*P*(*x*)) <0 and Im(*p*(*x*) = 0) (on the negative real axis), pick that one.
3. Otherwise, pick the unique *w*-mer *x* such that Im(*p*(*x*) < 0) and Im(*P*(R(*x*))) >0. *Intuitively, this is the w*-mer in the cycle right below the negative real axis.

This set breaks every pure cycle in the de Bruijn graph by its construction, with an interesting property:

**Lemma 8.** *Let* {*x_i_*} *be a path on the de Bruijn graph that avoids* ${ℳ}_{\sigma ,w}$. *If* Im(*P*(*x_i_*)) ≤0, *then for all j* ≥ 1, Im(*P*(*x_j_*)) ≤0.

*Proof.* It suffices to show that in the remaining de Bruijn graph after removing ${ℳ}_{\sigma ,w}$, there are no edges *x* → *y* such that Im(*P*(*x*)) ≤0 and Im(*P*(*y*)) >0. The edge *x* → *y* means that *y* = *S_a_*(*x*) for some *a*. By Lemma 7, Im(*P*(*R*(*x*))) = Im(*P*(*S_a_*(*x*))) = Im(*P*(*y*)) >0.

- If we have Im(*P*(*x*)) <0, by clause 3 of Definition 4, $x\in {ℳ}_{\sigma ,w}$.
- If we have Im(*P*(*x*)) = 0 and Re(*P*(*x*)) <0, by clause 2 of Definition 4, we have $x\in {ℳ}_{\sigma ,w}$.
- If we have Im(*P*(*x*)) = Re(*P*(*x*)) = 0, we would have Im(*P*(*y*)) = Im(*P*(*R*(*x*))) = 0, a contradiction.
- If we have Im(*P*(*x*)) = 0 and Re(*P*(*x*)) >0, *P*(*x*) lies on positive half of the real axis, so rotating it clockwise by 2π∕w degrees we would have Im(*P*(*y*)) = Im(*P*(*R*(*x*))) = 0, a contradiction.

### 3.4. Upper bounding the remaining path length in Mykkeltveit sets

In this section, we show that the remaining path after removing ${ℳ}_{\sigma ,w}$ is at most $O\left(w^{3}\right)$ long. This polynomial bound is a stark contrast to the number of remaining vertices after removing the Mykkeltveit set—that is, $\sigma^{w}-N_{\sigma ,w}∼\left(1-\frac{1}{w}\right)\sigma^{w}$, which is exponential in *w*. Our main argument involves embedding a *w*-mer to point in the complex plane, similar to Mykkeltveit's construction.

#### 3.4.1. From *w*-mers to embeddings

In this section, we formulate a relaxation that converts paths of *w*-mers to trajectories in a geometric space. Precisely, we model *S_a_* in Lemma 7 as a rotation operating on a complex embedding with attached weights, where the weights restrict possible moves.

Formally, given a pair (*z*, *t*) where *z* is a complex number and *t* an integer, define the family of operations $Z_{\delta }\left(z,t\right)=\left(r_{w}^{-1}z+\delta ,t+\delta \right)$. When z=P(x) is the position of a *w*-mer *x*, $t=W\left(x\right)=\sum_{i=0}^{w-1}x_{i}$ is its weight, and when $0\leq \delta +x_{0}<\sigma$, $Z_{\delta }\left(P\left(x\right),W\left(x\right)\right)=\left(P\left(S_{\delta +x_{0}}\left(x\right)\right),W\left(S_{\delta +x_{0}}\left(x\right)\right)\right)$. This means $Z_{\delta }$ is equivalent to finding the position and weight of the successor $S_{\delta +x_{0}}$.

We are now looking for the length of the longest path by repeated application of $Z_{\delta }$ that satisfies $0\leq t\leq W_{max}$, where $W_{max}=\left(\sigma -1\right)w$ is the maximum weight of any *w*-mer. This is a relaxation of the original problem of finding a longest path as some choices of δ and some pairs (z,t) on these paths may not correspond to the actual transition or *w*-mer in the de Bruijn graph (when $\delta +x_{0}$ is negative or greater than σ−1, then it is not a valid transition). In some sense, the pair (z,t) is a loose representation of a *w*-mer where the precise sequence of the *w*-mer is ignored and only its weight is considered. On the contrary, every valid path in the de Bruijn graph corresponds to a path in this relaxation, and an upper bound on the relaxed problem is an upper bound of the original problem.

#### 3.4.2. Weight-in embedding and relaxation

The *weight-in embedding* maps the pair x=(z,w) to the complex plane. This transforms the original longest remaining path problem into a geometric problem of bounding the length in the complex plane under some operation $S_{\delta }$.

**Definition 5** (Weight-In Embedding). The weight-in embedding of x=(z,t) is Q(x)=z−t. Accordingly, for a *w*-mer *x*, its embedding is Q(x)=Q(P(x),W(x))=P(x)−W(x).

The $Z_{\delta }$ operations in this embedding correspond to a rotation, and, maybe surprisingly, this rotation is independent of the value δ.

**Lemma 9.** *Let* x=(z,t). *For all* δ, *the point* $Q\left(Z_{\delta }\left(x\right)\right)$ *is the point* Q(x) *rotated clockwise* 2π∕w *around the point* (−t,0).

*Proof.* By definition of weight-in embedding and the operation $Z_{\delta }$:

$$Q\left(Z_{\delta }\left(z,t\right)\right)=r_{w}^{-1}z+\delta -\left(t+\delta \right)=r_{w}^{-1}\left(Q\left(z,t\right)+t\right)-t$$

In the complex plane, the rotation formula around center *c* and of angle *θ* is $c+e^{i\mathrm{Theta}}\left(z-c\right)$. Therefore, the operations $Z_{\delta }$ is a rotation around c=(−t,0) of angle Theta=−2π∕w.

Figure 2b shows the weight-in embedding of a de Bruijn graph. The set $S_{\sigma ,w}=\left\{\left(-j,0\right)|0\leq j\leq W_{max}\right\}$ is the set of all the possible centers of rotation, and is shown by large gray dots on the *x*-axis of Figure 2b. Because all the *w*-mers in a given conjugacy class have the same weight, say *t*_0_, the conjugacy classes form a circle around a particular center $\left(-t_{0},0\right)$. The image after application of $S_{\delta }$ is independent of the parameter δ, but dependent on the weight *t* of the underlying pair (z,t).

Multiple pairs of x=(z,t) can share the same weight-in embedding Q(x). As seen in Figure 2b, every node belongs to two circles with different centers, meaning there are two embeddings with the same Q(x) but different *t*.

Lemma 8 naturally divides any path in the de Bruijn graph avoiding ${ℳ}_{\sigma ,w}$ into two parts, the first part with Im(*P*(*x*)) >0, and the second part with Im(*P*(*x*)) ≥0. Thanks to the symmetry of the problems, we focus on the upper half-plane, defined as the region with Im(*P*(*x*)) ≥0. With the weight-in embedding, as long as the path is contained in the upper half-plane, it is always traveling to the right (toward large real value) or stays unmoved, as stated below:

**Lemma 10** (Monotonicity of Re(Q(·))). *Assume* Q(x) *and* $Q\left(Z_{\delta }\left(x\right)\right)$ *are both in the upper half-plane. If* Q(x) *does not coincide with its associated rotation center* (−t,0), *then* $Re\left(Q\left(Z_{\delta }\left(x\right)\right)\right)>Re\left(Q\left(x\right)\right)$, *otherwise* $Q\left(Z_{\delta }\left(x\right)\right)=Q\left(x\right)$.

*Proof.* The operation is a clockwise rotation where the rotation center is on the *x*-axis and the two points are on the non-negative half-plane. Necessarily, the real part increased, unless the point is on the fix point of the rotation [which is when Q(x)=(−t,0)].

We further relax the problem by allowing rotations from any of the centers in $S_{\sigma ,w}$, not just from some (−t,0) corresponding to the weight in the weight-in embedding. Lemma 10 still applies in this case and the points in the upper half-plane move from left to right. We are now left with a purely geometric problem involving no *w*-mers or weights to track:

What is the longest path $\left\{z_{i}\right\}$ possible where $z_{i+1}$ is obtained from *z_i_* by a rotation of 2π∕w clockwise around a center from $S_{\sigma ,w}$, while staying in the upper halfplane at all times ($Im\left(z_{i}\right)\geq 0,\forall i$)?

We now break the problem into smaller stages as the weight-in embedding pass through rotation centers, defined as $S_{\sigma ,w}=\left\{\left(-j,0\right)|0\leq j\leq W_{max}\right\}$, the set of points that Q(x) could possibly rotate around regardless of *t*. As there are $W_{max}+1$ rotation centers and the maximum Re(Q(x))=Re(P(x)) for any *w*-mer is also $W_{max}$, we define $2W_{max}$ subregions, two between any adjacent pair. Formally:

**Definition 6** (Half Subregions). A subregion is defined as the area $\left[-j,-j+0.5\right)\times \left[0,W_{max}\right]$ called a left subregion or $\left[-j+0.5,-j+1\right)\times \left[0,W_{max}\right]$ called a right subregion, for $0<j\leq W_{max}$.

We now define the problem of finding the longest path, localized to one left subregion, as follows:

**Definition 7** (Longest Local Trajectory Problem). Define the feasible region $\left(0,0.5\right)\times \left[0,W_{max}\right]$, and relaxed rotation centers $S\prime =\left\{\left(j,0\right)|-W_{max}\leq j\leq W_{max}\right\}$. A feasible trajectory is a list of points $\left\{z_{i}\right\}$ such that each point is in the feasible region, and *z_i_* can be obtained by rotating $z_{i-1}$ around c∈S′ clockwise by 2π∕w degrees. The solution is the longest feasible trajectory.

Again, note that this new definition is a purely geometric problem involving no *w*-mers and no weights W(x) to track. *z_i_* might stagnate if it coincides with one of the rotation centers, so we do not allow $Re\left(z_{i}\right)=-j$ in this geometric problem. Still, it suffices to solve this simpler problem, as indicated by the following lemma:

**Lemma 11.** *For fixed w and σ, if the solution to the problem in Definition 7 is L, the longest path in the de Bruijn graph avoiding* ${ℳ}_{\sigma ,w}$ *is upper bounded by* $4W_{max}L+O\left(w^{2}\right)=O\left(wL+w^{2}\right)$.

We prove this lemma in Supplementary Section S2.

#### 3.4.3. Backtracking, heights, and local potentials

In this section, we prove $L=O\left(w^{2}\right)$. We frequently switch between polar and Cartesian coordinates in this section and the next section. For simplicity, let r(z) and ϕ(z) denote the radius and the polar angle of *z* written in polar coordinate.

**Lemma 12**. *Any feasible trajectory within the region* $\left(0,d\right]\times \left[0,W_{max}\right]$ *for* d≤0.5 *is at most* $O\left(dw^{3}\right)$ *long.*

*Proof.* The key observation is if a rotation is not around the origin, Re(Q(x)) increases by $\Omega \left(1∕w^{2}\right)$.

To see this, assume (d,Theta) is the polar coordinate of Re(Q(x)) with respect to the rotation center. The polar coordinate for $Re\left(Q\left(S_{a}\left(x\right)\right)\right)$ is then (d,Theta−2π∕w). We note that d≥0.5 as Q(x) satisfies 0<Re(Q(x))<0.5 and is at least 0.5 away from any other rotation centers. The difference in real coordinate is d(cos(Theta−2π∕w)−cos(Theta))=2dsin(Theta−π∕w)sin(π∕w). Now, we require Theta∈[0,π] and Theta−2π∕w∈[0,π], so sin(Theta−π∕w)≥ sin(π∕w) and the whole term is lower bounded by $2d\sin^{2}\left(\pi ∕w\right)=\Omega \left(d∕w^{2}\right)=\Omega \left(1∕w^{2}\right)$.

Only $O\left(dw^{2}\right)$ rotations not around origin are possible in the defined region, otherwise Re(Q(x)) would increase by $\Omega \left(dw^{2}\right)\Omega \left(1∕w^{2}\right)=\Omega \left(d\right)$ already. Between two rotations not around the origin, only w∕2 rotations around the origin can happen, or the point would have rotated *π* degrees and cannot stay in the upper half-plane. This means the possible number of pure rotations is $O\left(dw^{3}\right)$, which is also the asymptotic upper bound of path length.

This lemma is sufficient to prove $L=O\left(w^{3}\right)$ and a total number of steps of $O\left(w^{4}\right)$. To obtain $L=O\left(w^{2}\right)$, we need a potential-based argument. Define $u=1-r_{w}^{-1}$, and let s(z) be the lowest point above the real axis of form z+ju where j∈Z. We can show that a potential function of form E(z)=−wr(s(z))∕π+ϕ(s(z)) is guaranteed to decrease by at least 2π∕w every rotation, and it can only decrease by O(w) total inside the feasible region, which would complete the proof. This proof can be found in Supplementary Section S3.

### 3.5. Lower bounding the remaining path length in Mykkeltveit sets

We provide here a constructive proof of the existence of a $\Omega \left(w^{2}\right)$ long path in the de Bruijn graph after removing ${ℳ}_{\sigma ,w}$. Since all *w*-mers in ${ℳ}_{\sigma ,w}$ satisfy Im(P(x))≤0, a path satisfying Im(P(x))>0 at every step is guaranteed to avoid ${ℳ}_{\sigma ,w}$ and our construction will satisfy this criterion. It suffices to prove the theorem for binary alphabet as the path constructed will also be a valid path in a graph with a larger alphabet. We present here the constructions for even values of *w*.

We need an alternative view of *w*-mers in this section, close to a shift register. Imagine a paper ring with *w* slots, labeled tag 0 to tag w−1 with content $y=y_{0}y_{1}\cdots y_{w-1}$, and a pointer initially at 0. The *w*-mer from the ring is $y_{j}y_{j+1}\cdots y_{w-1}y_{0}\cdots y_{j-1}=y\left[j,w-j\right]\cdot y\left[0,j\right]$, assuming pointer is at tag *j*. A pure rotation R(x) on the ring is simply moving the pointer one base forward, and an impure one $S_{a}\left(x\right)$ is to write *a* to *y_j_* before moving the pointer forward.

Let w=2m. We create ⌈w∕8⌉ ordered quadruples of tags taken modulo *w*: $Q_{j}=\left\{a-j,a+j,b-j,b+j\right\}$ where j∈[1,⌈w∕8⌉], a=m−1, and b=w−1. In each quadruple *Q_j_*, the set of associated root of unity $r_{w}^{i+1}$ for the four tags is of form $\left\{-e^{-i\mathrm{Theta}},-e^{i\mathrm{Theta}},e^{-i\mathrm{Theta}},e^{i\mathrm{Theta}}\right\}$, adding up to 0. Consequently, changing *y_k_* for each *k* in *Q_j_* from 1 to 0 does not change the resulting embedding. The strategy consists of creating “pseudoloops”: start from a *w*-mer, rotate it a certain number of times, and switch the bit of the *w*-mer corresponding to the index in a quadruple to 0 to return to almost the starting position (the same position in the plane but a different *w*-mer with lower weight).

More precisely, the initial *w*-mer *x* is all ones but $x_{w-1}$ set to zero, with paper ring content *y* = *x* and pointer at tag 0. The resulting *w*-mer satisfies P(x)=−1. The sequence of operations is as follows. First, do a pure rotation on *x*. Then, for each quadruple *Q_j_* from j=1 to j=⌈w∕8⌉, we perform the following actions on *x*: pure rotations until the pointer is at tag a−j, impure rotation *S*_0_, pure rotations until the pointer is at tag a+j, impure rotation *S*_0_, pure rotations until pointer is at tag b−j, impure *S*_0_, pure rotations until pointer is at tag b+j, impure *S*_0_.

**FIG. 3.**
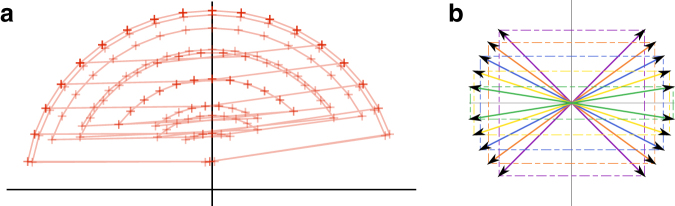
**(a)** For *w* = 40, each set of four arrows of the same color represents a quadruple set of root of unity. There are a total of five sets. They were crafted so that the four vectors in each set cancel out. **(b)** The path generated by these quadruple sets. The top circle of radius 1 is traveled many times (between tags *r*_1_ and *r*_2_ in each quadruple), as after setting the 4 bits to 0, the *w*-mer has the same norm as the starting point.

Each round involves exactly *w* + 1 rotations since the last step is to an impure rotation *S*_0_ at tag b+j, which increases by one between quadruples *Q_j_* and $Q_{j+1}$. The total length of the path over all *Q_i_* is at least $cw^{2}$ for some constant *c*. Figure 3 shows an example of quadruples and a generated long path that fits in the upper half-plane.

The correctness proof for the construction is presented in Supplementary Section S4 and the construction for odd *w* is presented in Supplementary Section S5.

## 4. Discussion

### 4.1. Relationship between UHS and selection schemes

Our construction of a UHS of relative size O(ln(w)∕w) and remaining path length *w* also implies the existence of a forward selection scheme with density O(ln(w)∕w), only a ln(w) factor away from the lower bound on density achievable by forward and local schemes.

Unfortunately, this construction does not apply for an arbitrary UHS. In general, given a UHS with relative size *d* and remaining path length *w*, it is still unknown how to construct a forward or a local scheme with density O(d). As described in Section 3.1, we can construct a UHS from a scheme by taking the set $S_{f}$ of contexts yielding new selections. However, it is not always possible to go the other way: there are UHS that are not equal to a set of contexts $S_{f}$ for any function *f*.

We are thus interested in the following questions. Given a UHS *U* with relative size *d*, is it possible to create another UHS U′ from *U* that has the same relative size *d* and corresponds to a local scheme (i.e., there exists *f* such that $U\prime =S_{f}$)? If not, what is the smallest price to pay (extra density compared to relative size of the UHS) to derive a local scheme from UHS *U*?

### 4.2. Existence of “perfect” selection schemes

One of the goals in this research is to confirm or deny the existence of asymptotically “perfect” selection schemes with a density of 1∕w, or at least O(1∕w). A study of UHS might shed light on this problem. If such a perfect selection scheme exists, asymptotic perfect UHS defined as (O(1∕w),w)-UHS would exist. On the contrary, if we denied the existence of an asymptotic perfect UHS, this would imply nonexistence of a “perfect” forward selection scheme with density O(1∕w).

### 4.3. Asymptotic results and practical uses of minimizer schemes

This line of research places more focus on asymptotic densities of minimizers (and naturally, asymptotic densities of local schemes, and asymptotic relative proportion and path length of UHS). That is, we focus on characterization of these quantities in the limit where *w* and *k*, the window length (number of *k*-mers in a window) and the length of a *k*-mer, go to infinity. On the contrary, for current practices, the values of *w* and *k* are relatively small, with *w* below 100 and *k* below 30 for the vast majority of use cases. While our results are not immediately useful to analyze these practical scenarios as we do not attempt to determine the constants behind the big-*O* notation, we believe that further refinement of our approaches can close the gap between theory and practice, and make rigorous analyses possible for practical minimizer and/or local schemes.

### 4.4. Remaining path length of minimum decycling sets

There is more than one decycling set of minimum size (MDS) for given *w*. The Mykkeltveit set (Mykkeltveit, 1972) is one possible construction, and a construction based on very different ideas is given in Champarnaud et al. (2004). The number of MDSs is much larger than the two sets obtained by these two methods. Empirically, for small values of *w*, we can exhaustively search all the MDSs on the binary alphabet: for 2≤w≤7 the number of MDSs is, respectively, 2, 4, 30, 28, 68 288, and 18 432.

While experiments suggest that the longest remaining path in a Mykkeltveit depathing set defined in the original article is around $\Theta \left(w^{3}\right)$, matching our upper bound, we do not know if such bound is tight across all possible minimal decycling sets. The Champarnaud set seems to have a longer remaining path than the Mykkeltveit set, although it is unknown if it is within a constant factor, bounded by a polynomial of *w* of different degree, or is exponential. More generally, we would like to know what is the range of possible remaining path lengths as a function of *w* over the set of all MDSs.

## Supplementary Material

Supplemental data

## References

[B1] Champarnaud, J.-M., Hansel, G., and Perrin, D. 2004. Unavoidable sets of constant length. Int. J. Algebra Comput. 14, 241–251

[B2] Chikhi, R., Limasset, A., and Medvedev, P. 2015. Compacting de Bruijn graphs from sequencing data quickly and in low memory. Bioinformatics 32, i201–i208 10.1093/bioinformatics/btw279 PMC4908363 27307618

[B3] DeBlasio, D., Gbosibo, F., Kingsford, C., et al. 2019. Practical universal K-mer sets for minimizer schemes, 167–176. In Proceedings of the 10th ACM International Conference on Bioinformatics, Computational Biology and Health Informatics, BCB’19. ACM, Niagara Falls, NY, New York, NY

[B4] Deorowicz, S., Kokot, M., Grabowski, S., et al. 2015. KMC 2: Fast and resource-Frugal k-Mer counting. Bioinformatics 31, 1569–1576 25609798 10.1093/bioinformatics/btv022

[B5] Golomb, S.W. 2014. Nonlinear shift register sequences, 110–168. In Shift Register Sequences. https://www.worldscientific.com/worldscibooks/10.1142/936. World Scientific. Singapore

[B6] Grabowski, S., and Raniszewski, M. 2013. Sampling the suffix array with minimizers, 287–298. In Iliopoulos, C., Puglisi, S., and Yilmaz, E, eds. String Processing and Information Retrieval. *Lecture Notes in Computer Science 9309.* Springer International Publishing. Switzerland

[B7] Jain, C., Dilthey, A., Koren, S., et al. 2017. A fast approximate algorithm for mapping long reads to large reference databases, 66–81. In Sahinalp, S.C., ed. *Research in Computational Molecular Biology*. *Lecture Notes in Computer Science*. Springer International Publishing. Switzerland 10.1089/cmb.2018.0036 PMC6067103 29708767

[B8] Li, H., and Birol, I. 2018. Minimap2: Pairwise alignment for nucleotide sequences. Bioinformatics 34, 3094–3100 29750242 10.1093/bioinformatics/bty191 PMC6137996

[B9] Lothaire, M. 2002. Algebraic Combinatorics on Words, vol. 90. Cambridge University Press. Cambridge, United Kingdom

[B10] Marçais, G., DeBlasio, D., and Kingsford, C. 2018. Asymptotically optimal minimizers schemes. Bioinformatics 34, i13–i22 29949995 10.1093/bioinformatics/bty258 PMC6037127

[B11] Marçais, G., Pellow, D., Bork, D., et al. 2017. Improving the performance of minimizers and winnowing schemes. Bioinformatics 33, i110–i117 28881970 10.1093/bioinformatics/btx235 PMC5870760

[B12] Marçais, G., Solomon, B., Patro, R., et al. 2019. Sketching and sublinear data structures in genomics. Annu. Rev. Biomed. Data Sci. 2, 93–118

[B13] Mykkeltveit, J. 1972. A proof of Golomb's conjecture for the de Bruijn graph. J. Comb. Theory Ser. B 13, 40–45

[B14] Orenstein, Y., Pellow, D., Marçais, G., et al. 2016. Compact universal K-mer hitting sets, 257–268. In Algorithms in Bioinformatics. *Lecture Notes in Computer Science.* Cham, Springer

[B15] Roberts, M., Hayes, W., Hunt, B.R., et al. 2004a. Reducing storage requirements for biological sequence comparison. Bioinformatics 20, 3363–3369 15256412 10.1093/bioinformatics/bth408

[B16] Roberts, M., Hunt, B.R., Yorke, J.A., et al. 2004b. A preprocessor for shotgun assembly of large genomes. J. Comput. Biol. 11, 734–752 15579242 10.1089/cmb.2004.11.734

[B17] Schleimer, S., Wilkerson, D.S., and Aiken, A. 2003. Winnowing: local algorithms for document fingerprinting, 76–85. In Proceedings of the 2003 ACM SIGMOD International Conference on Management of Data. SIGMOD’03. ACM. New York, New York, USA

[B18] Ye, C., Ma, Z.S., Cannon, C.H., et al. 2012. Exploiting sparseness in de novo genome assembly. BMC Bioinformatics 13, S1 10.1186/1471-2105-13-S6-S1 PMC3369186 22537038

