# Supplementary material for: Lower Density Selection Schemes via Small Universal Hitting Sets with Short Remaining Path Length
Source: J Comput Biol. 2021 Apr 20;28(4):395–409. doi: 10.1089/cmb.2020.0432 (PMC8066347; doi:10.1089/cmb.2020.0432)
Supplement: Supplemental data [file Supp_Data.docx]

## S1 Alternative Proof of Lemma 1

*Proof.* Assume there exists a path $\{c_{0},c_{1},\cdots,c_{w-1}\}$ in the de Bruijn graph of order $2w-1$ that avoids . As , there exists some indices $v$ such that $f(c_{w-1}[w-1,w])+(w-1)=f(c_{w-1}[v,w])+v$. Let *i* be the smallest $v$ satisfying this property.

As , there exists some indices *j* such that $f(c_{i}[w-1,w])+(w-1)=f(c_{i}[j,w])+j$. Now, note that $c_{i}[w-1,w]=c_{w-1}[i,w]$, so they have the same value of *f*. These two terms are underlined below.

$$\begin{matrix} f(c_{w-1}[w-1,w])+(w-1)=\underline{f(c_{w-1}[i,w])}+i \\ \underline{f(c_{i}[w-1,w])}+(w-1)=f(c_{i}[j,w])+j \\ 2(w-1)-i-j=f(c_{i}[j,w])-f(c_{w-1}[w-1,w])\leq w-1 \\ i+j-w+1\geq0 \end{matrix}$$

Let $t=i+j-w+1$. Since $i, j\in[0, w-2]$, $t=i+j-w+1<w-1$, so it is a valid index between $0$ and $w-2$. We now note that:

$$\begin{matrix} f(c_{w-1}[w-1,w])+(w-1)=f(c_{w-1}[i,w])+i \\ =f(c_{i}[w-1,w])+(w-1)+i-w+1 \\ =f(c_{i}[j,w])+j+i-w+1 \\ =f(c_{w-1}[t,w])+t \end{matrix}$$

Since $j < w-1$, we have $t < i$, contradicting with the fact that *i* is the smallest index satisfying $f(c_{w-1}[w-1,w])+(w-1)=f(c_{w-1}[v,w])+v$. □

## S2 Tightness of local trajectory problem

In this section, we prove Lemma 11 by resolving every difference between Definition 7 and the original problem of finding longest path avoiding . We start from the other type of subregions.

**Lemma S13.** *For any $0 \leq j < W_{max}$, any path in the de Bruijn graph avoiding has at most $2L+2$ steps satisfying $Q(x)\in(-j-1,-j) \times[0, W_{max}]$.*

*Proof.* As the defined region does not contain a rotation center, every move strictly increases $\text{Re}(Q(x))$. We similarly define the left and right subregion as the region with $\text{Re}(Q(x))<-j-0.5$ and $\text{Re}(Q(x))>-j-0.5$. There is at most one move that goes from the left subregion to the right one, or two moves if there is one $w$-mer with $Re(Q(x))=-j-0.5$, and all other moves are contained within either subregion.

For the left subregion, the longest path within it is upper bounded by *L*. Intuitively, we only need to shift the coordinate to coincide with Definition 7. Formally, let $\{z_{i}\}$ be the weight-in embedding of any path strictly within the left subregion. Then $\{z_{i}+(j+1)\}$ becomes a feasible trajectory under Definition 7, as all points are within the feasible region $(0,0.5) \times[0, W_{max}]$, and each center of rotation, which after shift is as $0\leq j<W(x)$.

For the right subregion, we have the same conclusion using a mirroring argument. To see this, again let $\{z_{i}\}$ be the weight-in embedding of any path strictly within second subregion. Then $\{j-\overline{z_{-i}}\}$ ($z_{-i}$ is the $i^{\text{th}}$ element in *z* counted backwards, and $\bar{z}$ is the conjugate of *z*) becomes a feasible trajectory. This is because all points are in the feasible region $(0,0.5) \times[0, W_{max}]$, and assuming $z_{i+1}$ is $z_{i}$ rotated $2\pi/w$ clockwise around $(-t, 0)$, we know $j-\overline{z_{i}}$ is $j-\overline{z_{i+1}}$ rotated $2\pi/w$ clockwise around . □

Next, we bound the path length outside any subregions.

**Lemma S14.** *Any path in the de Bruijn graph satisfying $Re(Q(x))>0$ and $Im(Q(x))\geq0$ has at most $w/4$ steps.*

*Proof.* We let *θ* denote the polar angle of $Q(x)$. As $Q(x)$ is in first quadrant, $0\leq\theta<\pi/2$. Next, observe that every rotation around the origin decreases *θ* by $2\pi/w$, and every rotation not around origin but some $(-i, 0)$ with $i > 0$ will decrease *θ* by a greater amount. This means the path is at most $w/4$ steps long, because in $w/4$ steps *θ* would have decreased by at least $\pi/2$, leading to a contradiction. □

We can similarly bound the path length left of all regions by looking at the polar angle of $Q(x)$ when origin is at $(-W_{\text{max}}, 0)$, which leads to the following lemma:

**Lemma S15.** *Any path in the de Bruijn graph satisfying $Re(Q(x))<-W_{max}$ and $Im(Q(x))\geq0$ has at most $w/4$ steps.*

We are now prove the bound over the entire upper halfplane by bounding path length on the boundaries of subregions.

**Lemma S16.** *Any path in the de Bruijn graph satisfying $Im(Q(x))\geq0$ has at most $2W_{max}L+O(w^{2})$ steps.*

*Proof.* We again start by taking $\{z_{i}\}$ to be the weight-in embedding of any path within the upper halfplane. We categorize $\{z_{i}\}$ using their real coordinates.

• If $Re(z_{i})<-W_{\text{max}}$ or $Re(z_{i})>0$, by previous two lemmas, we know there are at most $w/2$ of them.

• Else, if $Re(z_{i})$ is not an integer, it falls in one of the regions defined by Lemma S13. As there are $W_{\text{max}}$ regions total under that definition, and the point can never reenter a region, the point belongs to a path contained within the region of at most $2L+2$ length, and there are at most $W_{\text{max}}(2L+2)$ points in this category.

• The last category is when $Re(z_{i})$ is an integer. If $c_{i}$ satisfies $Im(z_{i})>0$, it will be the only one with this real coordinate as $Re(z_{i+1})>Re(z_{i})$. Otherwise, $c_{i}$ coincides with one of rotation centers $(-j, 0)$. It could stay at the same location by doing a rotation around itself, which corresponds to a pure rotation of a $w$-mer when that *w*-mer embeds to origin. By construction of (clause 1 of Definition 4), there can only be $w-1$ consecutive moves this way, so at most *w* elements in $\{z_{i}\}$ have this real coordinate. There are $W_{\text{max}}+1$ possible real coordinates, and each one of them might contain *w* points, so total number of points in this category is $(W_{\text{max}}+1)w$.

Summing these $3$ categories, we get a bound of $2W_{\text{max}}L+(W_{\text{max}}+1)w+w/2=2W_{\text{max}}L+O(w^{2})$ for a path in the upper-half plane. □

Finally, we look at the path in the lower halfplane with the concept of *w*-mer complements:

**Definition S8** (Complements of *w*-mer) *For $a\in\Sigma$, its complement is defined as $\bar{a}=\sigma-a$. For a $w$-mer $x$, its complement $\bar{x}$ is the $w$-mer $\overline{x_{0}}\overline{x_{1}}\cdots\overline{x_{w-1}}$. The following property holds:*

• *For any $x$,* $P(x)=-P(\bar{x})$.

• *For any $x$ and $a$,* $P(S_{a}(x))=-P(S_{\bar{a}}(\bar{x}))$.

• *If there is an edge* $x\to y$ *in the de Bruijn graph, there is also an edge* $\bar{x}\to\bar{y}$ *in the de Bruijn graph.*

**Lemma S17.** *Any path in the de Bruijn graph satisfying $Im(Q(x))\leq0$ has at most $2W_{\text{max}}L+O(w^{2})$ steps.*

*Proof.* For any path satisfying the condition, the path formed by taking complement of every *w*-mer is a path satisfying $Im(Q(x))\geq0$. By Lemma S16, the length is also upper bounded by $2W_{\text{max}}L+O(w^{2})$. □

We are now ready to prove the original statement as follows:

**Lemma 11.** *For fixed w and σ, if the solution to the problem in Definition 7 is L, the longest path in the de Bruijn graph avoiding*   *is upper bounded by* $4W_{max}L+O(w^{2})=O(wL+w^{2})$.

*Proof.* As seen in Lemma 8, we can bound the path length in two parts. For the first part with $Im(P(x))>0$, the length is upper bounded by $2W_{\text{max}}L+O(w^{2})$ because we prove a strictly stronger statement in Lemma S16 by also allowing points with $Im(P(x))=0$. For the second part with $Im(P(x))\leq0$, we also proved a strictly stronger statement in Lemma S17 by also allowing points in . The path length for the original problem is upper bounded by the sum of two upper bounds, which is $4W_{\text{max}}L+O(w^{2})$. □

## S3 Full Argument for $O(w^{3})$ Path Length Upper Bound

As mentioned in the main text, Lemma 12 does not solve our problems because setting $d=0.5$ yields $O(w^{3})$ long trajectories. We can however set $d=10/w$ and now focus on the trajectory in the region $(10/w, 0.5) \times[0, W_{\text{max}}]$, which we denote as for the rest of this section.

We aim to prove $L = O(w^{2})$ by proving the near-optimality of a greedy approach: The sequence of rotation such that $z_{i}$ only rotate around $(1,0)$ when necessary and otherwise rotate around $(0,0)$. Intuitively, if $z_{i}$ is rotated from further rotation centers, it will move a greater distance towards $Re(z_{i})=0.5$. For trajectories that include moves that deviate from the greedy trajectory, we want to show we can always backtrack to the move, make corrections and yield a longer trajectory. We now introduce the tools to formalize this idea.

We focus on the idea of backtracking moves. Recall the formula $z\leftarrow c+r_{w}^{-1}(z-c)$ for rotating *z* around *c* clockwise by $2\pi/w$ degrees. Note that this is a linear function of *c*, so if we change *c* by $(1,0)$, *z* will change by $u=1-r_{w}^{-1}$. In other words, if $z'$ is the result from rotating *z* around some centers, to change the rotation centers retroactively, we can simply move $z'$ by a multiple of *u*. This leads to the following definition.

**Definition S9** (Equivalence Classes and Heights). *Let $u=1-r_{w}^{-1}$ and recall . For any point , its equivalent set . The point with smallest j in the set is called representative of the set, denoted $s(z)$. The height of a point is defined as the nonnegative integer j such that $s(z)=z-ju$, which is zero if and only if* z *is a representative itself.*

Now we can define the potential function. Loosely speaking, this potential function measures how many steps are left in the trajectory if we strictly follow the greedy approach, backtracking one step if necessary.

**Definition S10** (Local Potential Function). *Let $P(z)=-wr/\pi+\varphi$, assuming $z=(r,\varphi)$ in polar coordinate. The potential function of a point is $E(z)=P(s(z))$, where $s(z)$ is representative of z.*

For , it is not guaranteed the representative $s(z)$ is in the same region. However, we have the following lemma:

**Lemma S18.** *If , $z'$ is obtained by rotating z one step according to the longest trajectory problem, then as long as , .*

*Proof.* By definition of the representative, $Im(s(z'))\geq0$. Also by definition of the equivalent set, $s(z')$ is also obtained by rotating *z* by some points on the real axis, clockwise by $2\pi/w$ degrees. As we have shown before, such move is guaranteed to increase $Re(z)$, so $Re(s(z'))>Re(z)>10/w$. To show , we only need $Re(s(z'))<0.5$ and $Im(s(z'))\leq W_{\text{max}}$. However, since $Re(s(z'))\leq Re(s(z))$ and $Im(s(z'))\leq Im(s(z))$, would imply . □

This means after one rotation in , or the first step in the trajectory, $s(z)$ is guaranteed to be in the same region and would stay in the region unless $z'$ is already out of the region, indicating end of trajectory. For the rest of our proofs, we assume for the whole trajectory.

Our goal from now on is to prove that $E(z)$ reduces by some amount each rotation. Assume a rotation brings *z* to $z'$. Note that if we only care about $E(z)$, the rotation center is irrelevant as $s(z')$ is the same, so we can assume every move is a rotation around origin and $z'=r_{w}^{-1}z$, possibly followed by a shift in multiples of *u* (which does not change $s(z')$).

If *z* and $z'$ are both of height 0, $s(z)=z$, $s(z')=z'$ and if $z=(r,\varphi)$ in polar coordinate, $z'=(r,\varphi-2\pi/w)$. This means $E(z)-E(z')=2\pi/w$ and the potential drops by $2\pi/w$, a constant value. If they are both of height $j$, $s(z)=z-ju$, $s(z')=z'-ju$, and it is no longer clear how much $\varphi$ changes other than that it decreases a bit. However, $z'$ is further to the origin, and as we prove below, the change in *r* is enough for our proofs. We prove a stronger lemma:

**Lemma S19.** *If $z'=e^{-i\theta}z$ is z rotated clockwise by $\theta\leq2\pi/w$ degrees, and they are of the same height $j\geq1$, then $E(z)-E(z')\geq1.9\theta$.*

*Proof.* We note that the $z'_{0}=z'-ju$ and $z_{0}=z-ju$ are the representatives of $z'$ and $z$, and $\varphi(z'_{0})<\varphi(z_{0})$. Let $r'=e^{-i\theta}$.

$$\begin{matrix} |z'_{0}|=|z'-ju| \\ =|r'z-ju| \\ =|r'(z_{0}+ju)-ju| \\ =|r'z_{0}+j(r'-1)u| \\ =|z_{0}+jr^{'-1}(r'-1)u| \end{matrix}$$

We let $y=r'^{-1}(r'-1)u$. Written in polar coordinate, $r'^{-1}=(1,\theta),(r'-1)=(2\sin(\theta/2),3\pi/2-\theta/2),u=(2\sin(\pi/w),\pi/2-\pi/w)$, so $y=(4\sin(\theta/2)\sin(\pi/w),\theta/2-\pi/w)$. Since , the polar angle of *y* is between 0 and $-\pi/w$, which becomes 0 as *w* grows, meaning *y* is almost parallel to real axis.

We next bound the polar angle of *z*_0_. Since *z*_0_ is in first quadrant, $\varphi(z_{0})\geq0$. Next, since and it is a representative, $Re(z_{0})\geq10/w$ and $Im(z_{0})\leq\sin(\pi/w)$ (otherwise, $z_{0}-t$ still satisfies $Im(\cdot)\geq0$ and would be the representative instead). For sufficiently large $w$, we have $Im(z_{0})\leq3/w$ and , which yields .

Let the angle between $z_{0}$ and *y* be $\psi$, we have . For large enough *w*, $|\psi| \leq0.3$ and $\cos(\psi)\geq0.955$. Apply the rule of cosines on vector additions:

$$\begin{matrix} |z'_{0}|^{2}=|z_{0}+jy|^{2} \\ =|z_{0}|^{2}+j^{2}|y|^{2}+2j\cos(\psi)|z_{0}||y| \\ \geq(z_{0}+0.955j|y|)^{2} \end{matrix}$$

So $r(z'_{0})-r(z_{0})\geq0.955j|y| = 3.82j\sin(\pi/w)\sin(\theta/2)\geq1.9\pi\theta/w$ for large enough *w*. We now plug this back to the formula for potential energy:

This finishes the proof. □

Plugging in $\theta=2\pi/w$, we have the following:

**Lemma S20.** *If* $z'=r_{w}^{-1}z$ *with same height* $h>0$, $E(z)-E(z')\geq3.8\pi/w$ *for sufficiently large* $w$.

The last case is when *z* and $z'$ are of different height. We need to account for the sudden change of height during rotation. Intuitively, changing height from $j+1$ to *j* while making a small movement costs $2\pi/w$ potential, as follows:

**Lemma S21.** *For sufficiently large w and a real number $10/w<d<0.5$, we have $P(d+u)-P(d)=-2\pi/w+O(1/w^{2})$.*

*Proof.* We will calculate the difference in $\varphi$ and *r* separately. Recall that $d=\Omega(1/w)$. For now, we let $u=a+bi$, that is, $a=2\sin^{2} (\pi/w), b=2\sin(\pi/w)\cos(\pi/w)=\sin(2\pi/w)$. For $\varphi$, we have:

For *r*, we have:

$$\begin{matrix} r(d+u)-r(d)=\sqrt{(d+a)^{2}+b^{2}}-d \\ =\sqrt{d^{2}+(a+b)^{2}+2ad}-d \\ =\sqrt{d^{2}+4\sin^{2} (\pi/w)(1+d)}-d \\ =\frac{4\sin^{2} (\pi/w)(1+d)}{\sqrt{d^{2}+4\sin^{2} (\pi/w)(1+d)}+d} \\ =\frac{4\pi^{2}(1+d)/w^{2}+O(w^{-4})}{2d+O(w^{-2})} \\ =\frac{2\pi^{2}(1+d)}{dw^{2}}+O(w^{-3}) \end{matrix}$$

Merging the two terms we have:

This finishes the proof.

Combining previous two lemmas, we can analyze the potential drop for all possible moves and prove the upper bound.

**Lemma S22.** *If* $z'=r_{w}^{-1}z$ *with different height, $E(z)-E(z')\geq3.9\pi/w$ for sufficiently large w*.

*Proof.* First of all, the height will only decrease since $z'$ is generated by rotating *z* around origin in the first quadrant, and $Im(z') < Im(z)$. For now, assume the height of *z* is *h* and height of $z'$ is $h-1$. We consider the movement of *s*(*z*) while rotating from *z* to $z'$. There exists one point $z''$ on the arc from *z* to $z'$ such that from *z* to $z''$ the height of the point is *h*, and from $z''$ to $z'$ the height is $h-1$. We can now divide the movement into three parts:

$$E(z)-E(z')=(E(z)-E(z''))+(E(z'')-E(z''+\delta))+(E(z''+\delta)-E(z'))$$

where $\delta$ is an infinitesimal value such that height of $z''$ is *h* and height of $z''+\delta$ is $h-1$. The first and last term correspond to the rotation process with constant height. If the height is 0, the change in potential is exactly the degree rotated. Otherwise, as shown in Lemma S19, the change in potential is at least 1.9 times degree rotated. Since the total rotated degrees is $2\pi/w$, these two terms add up to at least $2\pi/w$. The second term corresponds to the change of height as described in Lemma S21, and for sufficiently large *w*, it is at least $1.9\pi/w$. Adding both terms up, we get $3.9\pi/w$ as desired. We can use the same technique if the height drops more than 1 and yield at least the same bounds. □

**Lemma S23.** $L=O(w^{2})$ *as in Definition 7.*

*Proof.* We can divide the trajectory into two parts. The trajectory in the region $(0, 10/w) \times[0, W_{\text{max}}]$ is $O(w^{2})$ long as seen in Lemma 12. The potential function $E(z)$ has a maximum value of $\pi/2$ and minimum value of $O(w)$. The minimum holds because $r(s(z))=\sqrt{Re(s(z))^{2}+Im(s(z))^{2}}$ is upper bounded by a constant for $Re(s(z))<0.5$ and $Im(s(z))<1/w$ (otherwise $s(z)-ju$ for $j > 0$ would be the representative). As shown in previous lemmas, each move decreases $E(z)$ by $\Omega(1/w)$, so at most $O(w^{2})$ steps are possible in the region $(10/w, 0.5) \times[0,W_{\text{max}}]$. □

With Lemma 11, we conclude is a UHS with remaining path length $O(w^{3})$.

## S4 Correctness Proof for even *w*

The following lemma implies the created path avoids the Mykkeltveit set.

**Lemma S24.** *The sequence generated from the algorithm satisfies $Im(P(x))>0$ at every step.*

*Proof.* We define the *absolute embedding* as $P(y)$ on the paper ring model. This embedding does not change during pure rotations, and if $P(y)=(r,\varphi)$ in polar coordinate, the real embedding is $r_{w}^{-j}P(y)$ or in polar coordinate with pointer at tag $j$.

For each quadruple, we let $v_{i}=r_{w}^{r_{i}+1}$ denote the weight in the absolute embedding for tag $r_{i}$, where $1 \leq i \leq4$. We also let $\theta$ be the polar angle of $v_{4}$. As seen before, the absolute embeddings are $v_{1}=-e^{-i\theta}, v_{2}=-e^{i\theta}, v_{3}=e^{-i\theta}, v_{4}=e^{i\theta}$, and the corresponding polar angles are .

We now compute the change of $P(y)$, the absolute embedding, and polar angle of $P(x)$ (we use the phrase “phase” for it) throughout a round as in the following table:

| Stage | $P(y)$ Composition | $P(y)$ Polar Coordinate | Starting Phase | Ending Phase |
| --- | --- | --- | --- | --- |
| To Tag $r_{1}$ | $-1$ | $(1,\pi)$ | $\pi-\theta$ | $\theta$ |
| To Tag $r_{2}$ | $-1-v_{1}$ | $(2\sin(\theta/2), 3\pi/2-\theta/2)$ | $(\pi+\theta)/2$ | $(\pi-3\theta)/2$ |
| To Tag $r_{3}$ | $-1-v_{1}-v_{2}$ | $(2\cos(\theta)-1, 0)$ | $\pi-\theta$ | $\theta$ |
| To Tag $r_{4}$ | $-1-v_{1}-v_{2}-v_{3}$ |  | $(\pi+3\theta)/2$ | $(\pi-\theta)/2$ |
| End | $-1$ | $(1,\pi)$ | $\pi-\theta-2\pi/w$ |  |

Since $\theta$ increases by $2\pi/w$ each round, the ending condition for one round matches the starting condition for next round. Before the first round (as we do one pure rotation before first quadruple in the sequence of rotations), the polar angle is at $\pi-2\pi/w$, which matches the starting condition for round 1 with $\theta=2\pi/w$.

As long as $\theta< \pi/3$ (or in our constructions $i < w/6$), during all rotations the polar angle stays between 0 and *π*, meaning it stays strictly above the real line and thus avoids .

## S5 Construction for odd *w*

We focus on a particular portion of the path from last section with the property that all *w*-mers in the path are well above the real axis. We also define the set of *critical embeddings* for a round as the set of embeddings right before or after an impure rotation (or a write in the tape model). For a quadruple, given *θ*, the absolute embedding (defined in the proof of Lemma S24) and the polar angle of all critical embeddings can be read from the table above.

**Lemma S25.** *For sufficiently large $w = 2m$, the movement sequences defined above from $j=w/20$ to $j=w/10$ satisfies $Im(P(x)) > 0.05$ at every step.*

*Proof.* Note that the choice of *j* means $\theta$ is between $2\pi/10$ and $2\pi/20$. As pure rotations are arcs over upper halfplane, $Im(P(x))$ is the lowest at the endpoints of pure rotation, or as we defined above, the critical embeddings. However, at these points, the shortest embedding is $2\sin(\theta/2)>0.3$, and the smallest polar angle is *θ* with $\sin(\theta)>0.3$. We then have . □

Now let $w = 2m+1$ and again assume binary alphabet $\sigma= 2$. Let $a_{0}=m-1, a_{1}=m, b=w-1=2m$. The corresponding roots of unity (weights for tags in the absolute embedding) are $r_{w}^{b+1}=1$ for $b$, and $r_{w}^{a_{0}+1}$ and $r_{w}^{a_{1}+1}$ are the roots of unity directly above and below the vector $-1$. The starting $w$-mer is full 1 except $a_{0},a_{1}$ and *b* set to zero. The resulting absolute embedding is on the real axis with value $2\cos(\pi/w)-1=-1+4\sin^{2} (\pi/2w)=-1+O(w^{-2})$. We now construct a sequence of quadruples such that at the end of every quadruple the absolute embedding is still on the real axis with value close to $-1$.

We let *j* range from $w/20$ to $w/10$ as described before, but increment it by 2 every step.

**Definition S11** (Imperfect Quadruples) *For each j, we construct two candidate quadruples: $Q_{j}^{+}=\{a_{0}-j, a_{1}+j, b-j, b+j\}, Q_{j}^{-}=\{a_{0}-j+1, a_{1}+j-1, b-j, b+j\}$. Both quadruples satisfy that sum of their corresponding roots of unity is on real axis, which we denote as $q_{j}^{+}$ and $q_{j}^{-}$. We have $|q_{j}|=2(\cos(\theta+\pi/w)-\cos(\theta))=O(1/w)$ where $\theta$ is either $2\pi j/w$ or $2\pi j/w+\pi/w$, and are of opposite sign: $q_{j}^{+}>0,q_{j}^{-}<0$.*

*We define $l_{j}$ to be the embedding by setting all bases in quadruples $\{Q_{k}|k\leq j\}$ to zero from the initial $w$-mer. Our construction of the imperfect quadruples ensures $l_{j}$ is a real number.*

*We decide the imperfect quadruple to use depending on the sign of $l_{j-2}+1$. If $l_{j-2}$ is smaller than $-1$, we pick $Q_{j}^{-}$ and we have $l_{j}=l_{j-2}-q_{j}^{-}$. Otherwise, we pick $Q_{j}^{+}$ and we have $l_{j}=l_{j-2}-q_{j}^{+}$. In both cases, we assured $|l_{j}+1| \leq\max(|q_{j}|, |l_{j-2}+1|)$, which is $O(1/w)$ by induction on $j$.*

The sequence of rotations is defined in exactly the same way as before. The analyses are similar, as there are between $w+1$ and $w+3$ moves every round and $O(w^{2})$ total steps, no two quadruples share tags, and we finish the proof with the following lemma:

**Lemma S26.** *For every round of moves using imperfect quadruples, the embedding satisfies $Im(P(x))>0$ at all times.*

*Proof.* Similar to our previous argument, we only need to show $Im(P(x))>0$ at the critical embeddings. We start by constructing the following (perfect) quadruple for $2w$-mers: $Q'_{j}=\{w-1-2j, w-1+2j, 2w-1-2j, 2w-1+2j\}$. As seen in last lemma, the sequence generated by this quadruple satisfies $Im(P(x))>0.05$ at all times, so it also holds at the critical embeddings. We can also map the tags in $Q_{j}^{\pm}$ onto 2*w*-mers by keeping the corresponding roots of unity the same: $Q'_{j}^{+}=\{w-2-2j, w+2j, 2w-1-2j, 2w-1+2j\}$ and $Q'_{j}^{-}=\{w-2j, w-2+2j, 2w-1-2j, 2w-1+2j\}$.

Now we fix one embedding in the critical set. For example, at the end of writing 0 to tag $r_{3}$, the absolute embedding is $-1-v_{1}-v_{2}-v_{3}=-1+v_{4}$ and the polar angle is $(\pi+3\theta)/2$ for the perfect quadruple. We will prove that for the imperfect quadruple, the embedding at this moment is similar.

The absolute embedding is a combination of $v_{0}$ (which is $-1$ for the perfect quadruple, and $l_{j-2}=-1+O(1/w)$ for the imperfect one) and $\{v_{i}\}$s. $v_{3}$ and $v_{4}$ are the same for the two quadruples, while $v_{1}$ and $v_{2}$ are off by $\pi/w$ degrees, translating to $O(1/w)$ distance on the complex plane. This means the absolute embedding differs by $O(1/w)$.

The polar angle relative to the absolute embedding is simply one of the , which is off by at most $\pi/w$. This corresponds to an extra $\pi/w$ rotation in either direction, and since the length of the embedding is $O(1)$, it moves by $O(1/w)$ on top of the previous argument.

Combining both arguments, we show that if *z* is the embedding for the perfect quadruple at this moment, the embedding for the imperfect quadruple $z'$ satisfies $|z'-z|=O(1/w)$. However, $Im(z)>0.05$, so $Im(z')>0$ holds for sufficiently large *w*. This proof works for all critical embeddings, and since $Im(z')$ is the lowest at critical embeddings, $Im(z')>0$ also holds for the whole round. □
